# Expression of ICOSL is associated with decreased survival in invasive breast cancer

**DOI:** 10.7717/peerj.6903

**Published:** 2019-05-16

**Authors:** Bin Wang, Huayong Jiang, Tingyang Zhou, Ning Ma, Wei Liu, Yajie Wang, Li Zuo

**Affiliations:** 1 Department of Oncology, Changhai Hospital, the Second Military Medical University, Shanghai, China; 2 Department of Radiation Oncology, The 7th Medical Center of PLA General Hospital, Beijing, China; 3 Radiologic Sciences and Respiratory Therapy Division, School of Health and Rehabilitation Sciences, The Ohio State University College of Medicine, Columbus, OH, USA; 4 Interdisciplinary Biophysics Graduate Program, Ohio State University, Columbus, OH, USA; 5 Clinical Laboratory, 905th Hospital of PLA, Shanghai, China; 6 Department of Radiation Oncology, Mayo Clinic Arizona, Pheonix, AZ, USA; 7 College of Arts and Sciences, University of Maine Presque Isle Campus, ME, USA

**Keywords:** ICOSL, Prognosis, Invasive breast cancer

## Abstract

**Background:**

Inducible co-stimulator (ICOS) is a CD28-related molecule exclusively expressed on activated T cells and plays a critical role in modulating the immune response in breast cancer. The blockage of ICOS pathway has been shown to inhibit the activity of Type 2 T helper cells, thus potentially protecting against cancer growth. The current study aims to investigate the correlation between inducible co-stimulator ligand (ICOSL) expression in tumor tissues and the prognoses of patients with invasive breast cancer.

**Methods:**

Tumor samples from 562 Chinese patients with invasive breast carcinomas were collected between 2003 and 2010. The expression of ICOSL on breast tumor and adjacent non-cancerous tissue was determined via immunohistochemistry. The overall survival (OS) of patients with positive and negative ICOSL expression were described using Kaplan–Meier curves, respectively. Parametric correlation method was used to analyze the correlation between ICOSL expression and other clinicopathological parameters. ICOSL was selected as a dependent variable for multivariate analysis.

**Results:**

Positive ICOSL expression was identified on the plasma membrane in both cytoplasm and the nucleus of breast cancer cells. Membrane-expressed ICOSL is determined as an independent prognostic factor for OS in breast cancer but without significantly correlating with other clinicopathologic parameters such as age, menopausal status, depth of invasion, lymph node metastasis status, histologic classification, etc.

**Conclusion:**

Our study suggests that the up-regulated expression of ICOSL protein in breast tumor cells can be associated with poor prognoses in invasive breast carcinomas.

## Introduction

Breast cancer is the second leading cause of cancer-related death among US women (DeSantis et al., 2011). Research has shown that immune activity plays a key role in breast cancer progression and it has become increasingly clear that cancer cells can evade immune surveillance via the regulation of immune reactivity (Cavallo et al., 2011; DeNardo & Coussens, 2007). For example, cytotoxic T lymphocytes and type-1 T helper (Th1) cells respond positively to protect against tumor development. However, Th2 cells and humoral immunity can be activated to promote cancer progression (DeNardo & Coussens, 2007). It is of primary importance to understand the regulatory mechanism of tumor-associated immune cells in order to develop effective treatment therapies. The activation of T cells requires two steps of signaling between T cells and antigen-presenting cells (APCs): the first step involves the binding of T-cell receptor with antigen-major histocompatibility complex on APCs; and the second step involves the binding of CD28, a T cell co-stimulatory protein, to its counter receptor B7-1 on APCs (Hutloff et al., 1999; Wallin et al., 2001).

Inducible co-stimulator (ICOS) is a CD28-related molecule exclusively expressed on activated T cells (Hutloff et al., 1999; Wallin et al., 2001). Its counter ligand inducible co-stimulator ligand (ICOSL) can be found in professional APCs such as dendritic cells (DCs), B lymphocytes, as well as some cancer cells (e.g., implanted-transfected tumors) (Aspord et al., 2013; Le et al., 2016; Martin-Orozco et al., 2010; Schreiner et al., 2003; Wang et al., 2012). ICOSL demonstrates a positive co-stimulatory effect by specifically modulating the responses from regulatory T cells (Treg) and Th2 cells (Akbari et al., 2002; Vieira et al., 2004). The blockade of ICOSL significantly reduced the generation of Th2 cytokines including IL-4 and IL-10, but did not affect the levels of the Th1 cytokines such as IFN-γ and IL-2 (Aspord et al., 2013; Wang et al., 2012). Furthermore, ICOS-positive T cell infiltration was reported to be negatively associated with patient prognosis in breast cancer (Faget et al., 2013).

The relationship between cancer development and tumor-related ICOSL expression remains controversial. It was previously reported that ICOSL expression in solid tumors aids in the activation of CD8^+^ cytotoxic T cells, thus triggering anti-tumor responses (Flies & Chen, 2007). Similarly, several studies using ICOSL transfected solid tumor cell lines showed that ICOSL contributes to tumor regression via CD8^+^ cytotoxic lymphocyte-mediated pathways (Liu et al., 2001; Wallin et al., 2001). Conversely, Tamura et al. (2005) suggested that a shorter survival time of patients was associated with the expression of ICOSL in acute myeloid leukemic cells. Therefore, further studies are needed to determine the roles of ICOSL in mediating immune responses in the context of various cancers. Specifically, the expression of ICOSL in breast cancer has not been elucidated. The present study was designed to analyze the prognostic significance of ICOSL protein expression in patients with invasive breast cancer cases. Our results may contribute important insights to further the study of ICOSL in tumor cells and provide possible treatment recommendations.

## Materials and Methods

### Study population

A total of 562 patients with breast cancer were included in the present study. They were diagnosed with invasive breast cancer and had surgery between January 2003 and December 2010 at the First Affiliated Hospital of Second Military Medical University (Changhai Hospital, Shanghai, China). After surgery, paraffin-embedded pathologic specimens of the tumor and adjacent noncancerous tissues were obtained and stored for later tissue microarray (TMA). Tumor-node-metastasis (TNM) classification (American Joint Committee on Cancer (AJCC)) was used in reference in order to classify each tumor. Patients in stage IV who had other primary tumor sites with a noncurative resection, or patients who underwent preoperative radiotherapy or chemotherapy were not included in the sample of 562 patients. Primary treatment was determined according to the guidelines of the National Comprehensive Cancer Network and the guidelines for diagnosis and management of breast cancer issued by the National Health and Family Planning Commission of the People’s Republic of China (http://www.nhc.gov.cn/). (Gradishar et al., 2014). In brief, the guidelines recommend the use of taxane plus anthracycline-based therapy for patients with lymph node metastasis, anthracycline-based therapy for patients without nodal metastasis, trastuzumab for patients with human epidermal growth factor receptor 2 (HER2)-positive cancer, and antihormonal treatment for patients with estrogen-receptor (ER)-positive cancer. The median age of the 562 patients was 53 years old (range: 31–85 years). The following variables were recorded: patients’ age at diagnosis, largest tumor diameter, menopausal status, number of lymph node metastasis, histologic type, histology grade (Elston-Ellis grade), TNM stage (AJCC), and the expression of ER, progesterone (PR), and HER2. Among those 562 patients, 120 of them were followed up. The median follow-up duration was 77 months in the present study (range, 3–115 months) and the overall survival (OS) was then recorded. All consent form and study procedures were approved by Ethics Committee of Changhai Hospital charged with ethical review of proposed research with human subjects (protocol #20140310).

### Tissue microarray and immunohistochemistry

A total of 562 pathologic specimens and 162 adjacent noncancerous tissue samples were obtained by surgical resection from patients. All these resection samples were treated using the standard protocol of fixation, dissection, and processing for TMA analysis. TMA blocks were constructed as follows: a tissue-arraying instrument was used to punch out 1.5 mm-diameter cylinders of the tumor center as the representative areas of a tissue block (Beecher Instruments, Sun Prairie, WI, USA). The areas of ulceration and necrosis were excluded. Punched tissues were re-embedded into a recipient paraffin block in defined positions. TMA blocks were cut into four mm sections for immunohistochemistry (IHC).

The specimens were washed with phosphate-buffered saline and then treated with the primary antibody using the following procedure: ICOSL antibody (dilution 1:50), ER antibody (dilution 1:50), PR antibody (dilution 1:50), HER2 antibody (dilution 1:50), respectively. All antibodies were purchased from Abcam (Cambridge, UK). Immunostaining was performed via the Envision System with diaminobenzidine (Dako, Glostrup, Denmark). For negative control, the primary antibody was replaced by a normal rabbit or murine immunoglobulin G with the same dilution.

### IHC evaluation

Two researchers (Bin Wang and Ning Ma), who were blinded to the clinical pathological data of the patients, evaluated the expression of ICOSL in the TMAs independently. Disagreements were resolved through discussions held between the two evaluators. According to the HER scoring system of 2013 ASCO/CAP guideline recommendations, HER2 “positive” was defined by IHC staining of +++ or ++ based on the fluorescence in situ hybridization result (Tchrakian et al., 2016). The expression of ICOSL, ER, and PR was graded by the two independent observers using a four-point scale, where 0 represents no staining, + represents light staining, ++ represents moderate staining, and +++ represents strong staining. Slices with a score of + were classified as having “low expression,” ++ “high expression,” and +++ “overexpression.” Tissues scored with +, ++, and +++ are defined as “positive.” By contrast, tissues scored with 0 are classified as having “no expression” or “negative.”

### Statistical analysis

A Chi-square test was used to determine the possible associations between the ICOSL expression and the clinic pathologic parameters. The effect of ICOSL expression on OS was expressed using Kaplan–Meier curves. The log-rank test was used to compare different survival curves. Multivariate proportional Cox models were employed to evaluate the prognostic significance of the expression of ICOSL, histological types, age, tumor stages, and the expression of HER2, ER, and PR. *P*-value ≤ 0.05 was considered significant. Statistical analysis was performed using SPSS19.0 software (SPSS Inc., Chicago, IL, USA).

## Results

### Expression of ICOSL in breast cancers

**Figure 1:**
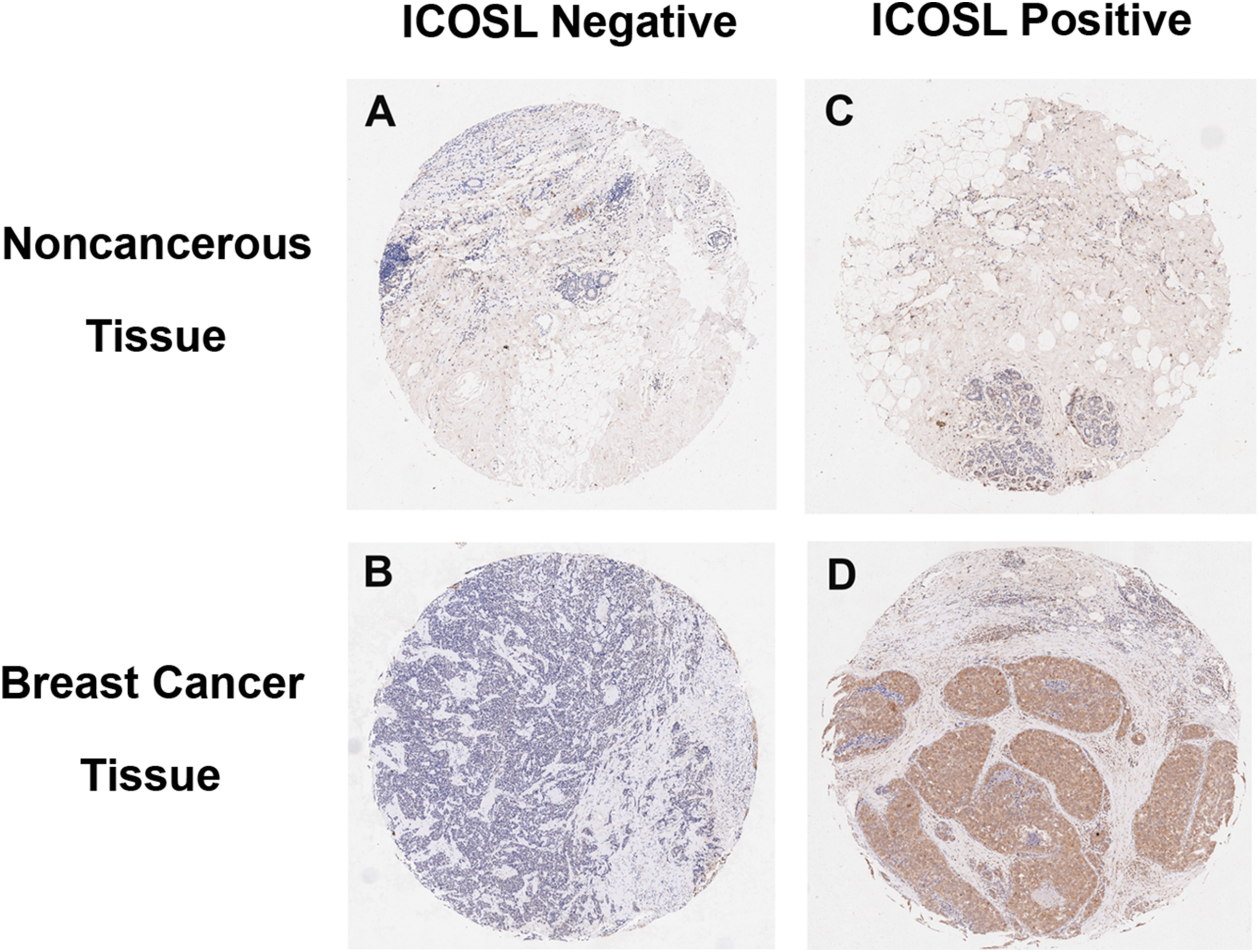
Representative images of breast cancer immunostaining for ICOSL in cancerous and noncancerous tissues (10× magnification). (A) Negative for adjacent noncancerous tissues. (B) Negative for adjacent cancerous tissues. (C) Positive for adjacent noncancerous tissues. (D) Positive for breast cancer tissues. ICOSL staining was shown as the brown color in the images.

**Figure 2:**
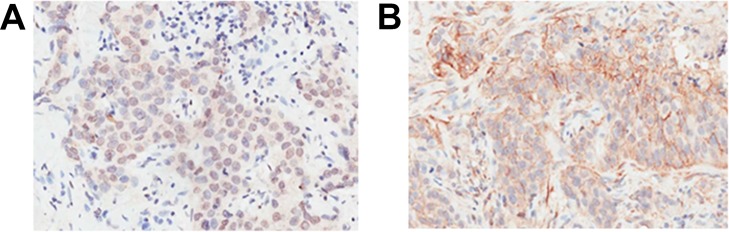
Representative images of breast cancer immunostaining for ICOSL at subcellular locations (200× magnification). (A) ICOSL was expressed primarily in the nucleus or cytoplasm. (B) ICOSL was expressed primarily in the cytoplasm or on the plasma membrane. ICOSL staining was shown as the brown color in the images.

Inducible co-stimulator ligand was expressed not only on the plasma membrane, but also in the cytoplasm and nucleus of cancer cells (Figs. 1 and 2). Positive ICOSL staining was found on the membrane in 44 (7.83%), in the cytoplasm in 370 (65.83%) and in the nucleus in 441 (78.47%) of all 562 examined carcinoma tissue samples. ICOSL staining was also detected in 162 adjacent non-cancerous tissues, which was compared with the staining of the corresponding cancerous tissues (Table 1). The results show that ICOSL expression was significantly higher in the cancerous tissues than in non-cancerous tissues either on the membrane, in the cytoplasm or in the nucleus (*P* = 0.042, *P* < 0.001, and *P* < 0.001, respectively) (Table 1). Since ICOS is exclusively expressed on the membrane of T cells, its interaction with ICOSL, located on cancer cell membranes, may be of pathological relevance in mediating immune cell function. Therefore, we focused on investigating the significance of membrane-expressed ICOSL in breast cancer.

**Table 1:** ICOSL expression in carcinoma vs. precancerous tissues.

	*N*	Carcinoma tissue		Precancerous lesions		Carcinoma–Precancerous lesions		
		Positive rate (%)	Score ${\bar {\rm X}}$	Positive rate (%)	Score ${\bar {\rm X}}$	${\bar {\rm X}}$	95% CI	*P*
Membrane	162	9.26	0.38	6.79	0.14	0.24691	[0.00914–0.48469]	0.042
Cytoplasm	162	81.48	4	38.27	1.37	2.62963	[2.07791–3.18135]	0
Nucleus	162	79.63	2.27	47.53	1.11	1.16049	[0.80069–1.5203]	0

**Note:**

CI, confidence interval; ICOSL, inducible co-stimulator ligand.

### Association between membrane-expressed ICOSL and clinicopathologic parameters

The correlation between ICOSL expression and specific clinicopathologic parameters was presented in Table 2. The results showed that levels of ICOSL protein expression on cell membrane was not significantly associated with these clinicopathologic parameters, which include age, menopausal status, depth of invasion, lymph node metastasis status, histologic classification, histologic type, and AJCC stage, etc. (*P* > 0.05).

**Table 2:** Correlations between ICOSL expression and clinicopathologic parameters.

	*N*	Negative (%)	Positive (%)	*P*
Age (Y)				
<60	413	386 (93.46)	27 (6.54)	0.253
≥60	149	132 (88.60)	17 (11.41)	
Menopause				
No	254	236 (92.91)	18 (7.08)	0.522
Yes	308	282 (91.56)	26 (8.44)	
Depth of invasion				
T1	209	191 (91.38)	18 (8.62)	0.397
T2/T3/T4	353	327 (92.63)	26 (7.37)	
Lymph node metastasis				
Negative (N0)	191	181 (94.76)	10 (5.24)	0.11
Positive(N1/N2/N3)	371	337 (90.84)	34 (9.16)	
Stage				
I	91	86 (94.51)	5 (5.49)	0.359
II/III/IV	471	432 (91.72)	39 (8.28)	
Histologic grade				
I/II	357	328 (91.88)	29 (8.12)	0.384
III	205	190 (92.68)	15 (7.32)	
Histologic type				
Ductal	497	460 (92.56)	37 (7.44)	0.373
Lobular	65	58 (89.23)	7 (10.77)	
ER				
Negative	238	217 (91.18)	21 (8.82)	0.603
Positive	324	301 (92.90)	23 (7.10)	
PR				
Negative	325	295 (90.77)	30 (9.23)	0.28
Positive	237	223 (94.09)	14 (5.91)	
HER2				
Negative	296	278 (93.92)	18 (6.08)	0.159
Positive	266	240 (90.22)	26 (9.78)	

**Note:**

ER, estrogen receptor; HER2, human epidermal growth factor receptor 2; PR, progesterone receptor.

### Effects of membrane ICOSL expression on overall survival

**Figure 3:**
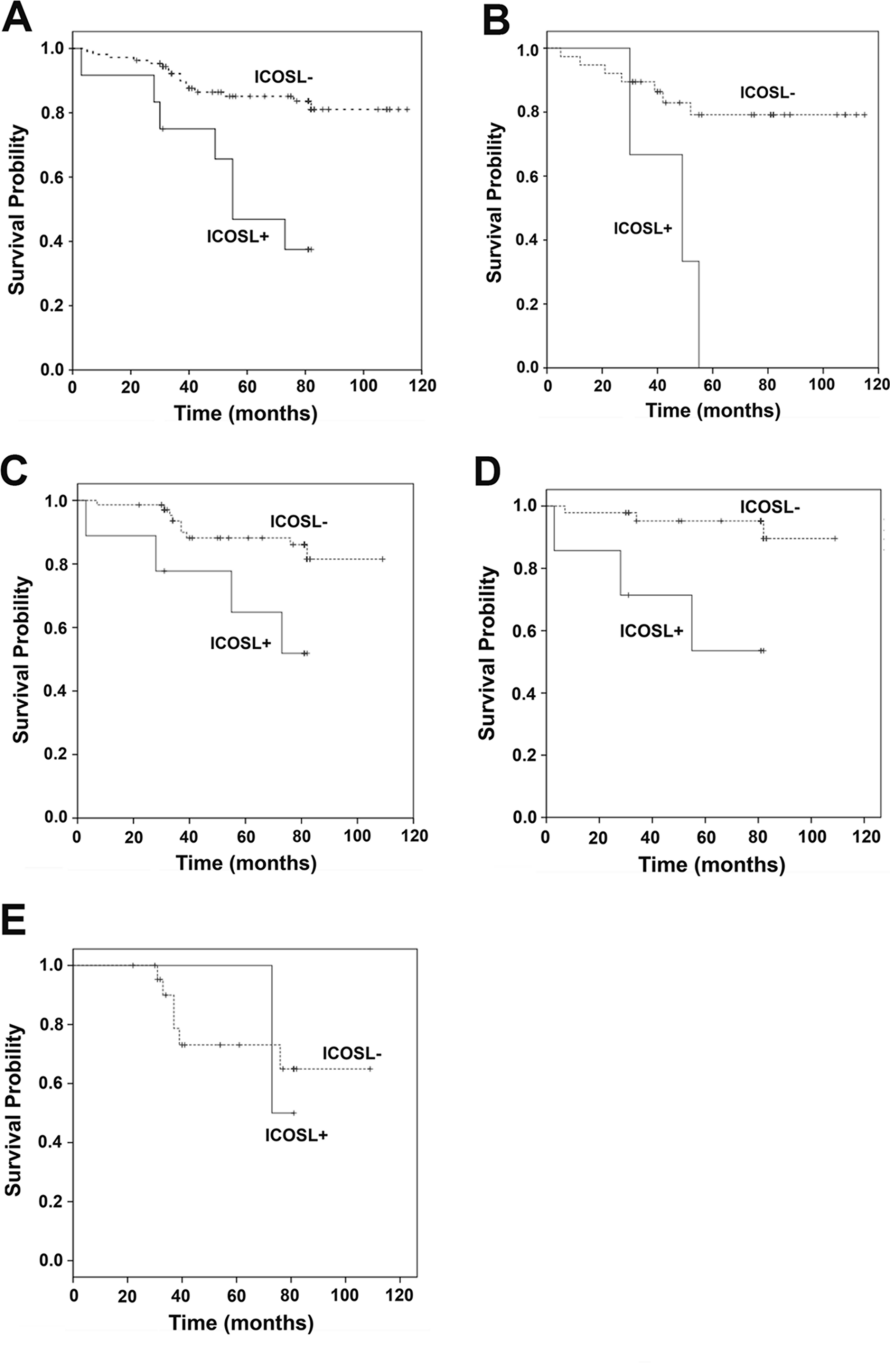
Kaplan–Meier curves for the overall survival of breast cancer patients in relation to ICOSL expression. (A) Kaplan–Meier curve of the overall survival in relation to ICOSL expression level in all the 121 patients (ICOSL negative 109 and 12 ICOSL positive, *P* = 0.000); (B) Kaplan–Meier curve of overall survival in relation to ICOSL expression level in all the 42 triple negative breast cancer subjects (ICOSL negative 39 and ICOSL positive 3, *P* = 0.003); (C) Kaplan–Meier curve of overall survival in relation to ICOSL expression level in all the 79 non-triple negative breast cancer subjects (ICOSL negative 70 and ICOSL positive 9, *P* = 0.012); (D) Kaplan–Meier curve of overall survival in relation to ICOSL expression level in all the 53 Luminal A/B breast cancer subjects (ICOSL negative 46 and ICOSL positive 7, *P* = 0.001); (E) Kaplan–Meier curve of overall survival in relation to ICOSL expression level in all the 25 HER2(+) breast cancer subjects (ICOSL negative 23 and ICOSL positive 2, *P* = 0.826).

To determine the potential impact of membrane ICOSL expression on patient survival, the Kaplan–Meier (K–M) procedure was applied according to different types of breast cancers such as Luminal A/B, HER2 positive and triple negative breast cancer (TNBC). Univariate analysis revealed that the patients with positive ICOSL in their tumors had significantly lower survival outcomes (*P* = 0.000, Fig. 3A). It was also found that patients with positive ICOSL in the tumors had poorer survival outcomes in TNBC and non-TNBC (*P* = 0.003, *P* = 0.012 respectively, Figs. 3B and 3C), as well as in luminal A/B breast cancer (*P* = 0.001, Fig. 3D), but not in patients with HER2 positive breast cancer (*P* = 0.826, Fig. 3E). A multivariate proportional hazard model was used to evaluate the prognostic relevance of the expression of ICOSL after being adjusted for the following clinical prognostic factors: age, AJCC cancer stage, histologic type, histology grade ER, progesterone (PR), and HER2 (Table 3). The results suggested that ICOSL is an independent prognostic factor of OS in breast cancer (ICOSL expression: HR, 1.353; 95% CI [1.165–1.572]; *P* = 0.000).

**Table 3:** Multivariate Cox proportional hazard regression modal analysis.

	*B*	SE	Wald	*df*	Sig.	Exp (B)	95.0% CI for Exp (B)	
							Lower	Upper
Age	0.040	0.021	3	1.000	0.063	1	0.998	1.085
Stage	0.828	0.448	3.420	1	0.064	2.290	0.952	5.509
Histologic type	0.119	0.726	0.027	1	0.869	1.127	0.272	4.676
Histologic grade	0.270	0.486	0.309	1	0.578	1.310	0.505	3.400
HER2	0.055	0.226	0.060	1	0.806	1.057	0.679	1.645
ER	0.046	0.255	0.033	1	0.855	1.048	0.636	1.727
PR	0.460	0.477	0.930	1	0.335	0.631	0.248	1.608
ICOSL	0.302	0.076	15.622	1	0.000	1.353	1.165	1.572

**Note:**

ER, estrogen receptor; HER2, human epidermal growth factor receptor 2; ICOSL, inducible co-stimulator ligand; PR, progesterone receptor.

## Discussion

To the best of our knowledge, this is the first study to investigate the prognostic significance of tumor-associated ICOSL expression in the patients with breast carcinomas. ICOSL expression showed markedly higher levels of expression than adjacent non-cancerous tissues. Membrane-expressed ICOSL was correlated with a poor survival rate in TNBC, non-TNBC, luminal A/B breast cancers and was determined as an independent prognostic factor for breast carcinoma. Interestingly, no association was found between ICOSL expression and the prognosis of HER2 positive patients.

Human epidermal growth factor receptor 2 overexpression is associated with the invasive phenotype and low survival rate in breast cancer (Ayoub, Al-Shami & Yaghan, 2019). HER2 is a transmembrane receptor, the activation of which triggers PI3K/Akt and Ras/MAPK signaling pathways, contributing to tumor cell survival and proliferation (Ayoub, Al-Shami & Yaghan, 2019; Valabrega, Montemurro & Aglietta, 2007). Furthermore, HER2(+) breast cancer was reported to have a higher proportion of CD4^+^ T cells and Treg than the luminal breast cancer subtype (Meng et al., 2018). Similarly, ICOSL expression has a positive modulatory effect on Treg and Th2 cells which suppress the immune activity (Faget et al., 2012). Therefore, positive ICOSL expression can potentially aggravate the prognosis of patients with HER2(+) breast cancer. However, our results showed that the HER2(+) patient with positive ICOSL had a similar survival rate to the patients with negative ICOSL. This is possibly because that the immune environment of HER2(+) breast cancer was not significantly altered by the expression of ICOSL on tumor cell membrane. To test this hypothesis, further study is needed to examine the profiles of immune cells (e.g., the levels of CD4^+^ cells and Treg) in HER2(+) breast cancer with positive and negative ICOSL expression, respectively.

Breast tumors are commonly infiltrated by Treg, memory CD4^+^ T cells, and DCs (Faget et al., 2012). ICOS positive CD4^+^ cells and tumor-associated Treg can be activated by ICOSL-expressed plasmacytoid DCs (Faget et al., 2012). The activation of the ICOS pathway plays a key role in activating the expansion of Treg and the release of IL-10 by secretion from memory CD4^+^ T cells, both of which create an immunosuppressive environment for cancer growth (Akbari et al., 2002; Faget et al., 2013; Gobert et al., 2009; Sharma et al., 1999). It has been reported that ICOS positive immune cell infiltration is linked with poor prognosis of breast cancer and accounts for a strong predictor of disease (Conrad et al., 2012; Faget et al., 2013). ICOSL is typically located on APCs as well as in specific cancer cells such as glioma cells, melanoma, and hematologic neoplasm cells (Aspord et al., 2013; Martin-Orozco et al., 2010; Schreiner et al., 2003; Wang et al., 2012). ICOSL-expressed plasmacytoid DCs have been correlated with an increased regulatory immune response in melanoma (Aspord et al., 2013). Martin-Orozco et al., for instance, identified ICOSL expression on the cell surface of melanomas. High levels of ICOSL expression was found in 50% of metastatic melanoma samples (Martin-Orozco et al., 2010). Also, ICOSL-expressed by melanoma cells may induce the increased ICOS expression in Treg. As a result, tumor cells can operate as APCs, promoting Treg expansion by themselves (Ito et al., 2008; Martin-Orozco et al., 2010). Our previous study on mouse hematologic neoplasm cells indicated that a blockade of ICOS/ICOSL pathways in tumor cells led to the downregulation of IL-4 and IL-10, both of which have anti-inflammatory effects (Wang et al., 2012). The current study identified for the first time the expression of ICOSL in breast cancer cells on the membrane, in the cytoplasm, as well as in the nucleus. Membrane-expressed ICOSL is significantly associated with a poor OS of breast carcinoma. Therefore, the expression of ICOSL on the plasma membrane potentially endows breast cancer cells with the ability to modulate immune activity. The activation of ICOSL/ICOS signaling may trigger the Th2-type responses, and thus negatively regulates the immune cell responses against tumors, which is responsible for the poor survival in breast cancer patients (Wang et al., 2013). The functional roles of ICOSL expressed in acute myeloid leukemia (AML) has been reported by Han et al. (2018) who showed that ICOSL expressed by AML cells can directly drive the expansion of Treg and secretion of IL-10. However, it remains unclear whether ICOSL-expressed breast cancer cells can directly interact with CD4^+^ T cells as a mechanism of immune suppression, contributing to the poor OS observed in our study. Therefore, one of our immediate tasks is to explore the mechanisms underlying the potential interactions between ICOSL-overexpressed breast cancer cells and tumor-associated T cells.

The prognostic relevance of the expression of ICOSL was assessed using a multivariate proportional hazard model. We found that membrane-expressed ICOSL in tumor cells is an independent prognostic factor for breast cancer. Traditional prognosis factors for breast cancer include tumor size, grade, and status of lymph node, which constitute the Nottingham Prognostic Index (NPI). Patients with the lowest NPI score had a 10-year survival rate of 85%. The mitotic activity index, which indicates cancer proliferative activity and lymphovascular invasion are both suggested as accurate prognosis factors for long-term survivals (Soerjomataram et al., 2008). Other markers such as the p53 mutation and HER2 have recently been identified to be associated with poor prognostic results in specific breast cancer patients (Soerjomataram et al., 2008). Our data suggested membrane-expressed ICOSL could be a novel and independent prognostic factor for invasive breast cancer. Significant poor OS was found among the patients with positive ICOSL on tumor cell membrane for TNBC and non-TNBC, as well as luminal A/B breast cancer. Further research is needed to establish the quantitative relationship between ICOSL expression and OS by including larger sample sizes. It would also be of primary interest to investigate the therapeutic potential of inhibiting ICOSL pathways to treat invasive breast cancer in future studies.

Furthermore, PD-L1 and PD-L2, similar to ICOSL, are members of B7 family of immune-regulatory ligands. PD-L2 has its expression restricted to lymphoid tissues, whereas PD-L1 and ICOSL can be expressed in lymphoid and non-lymphoid tissues (Collins, Ling & Carreno, 2005). PD-L1 is significant because substantial amounts have been found on many murine and human cancer cells, including breast cancer (Blank, Gajewski & Mackensen, 2005). PD-L1 and PD-L2 have been identified as ligands for the receptor PD-1, which is expressed on CD4, CD8, B cells, monocytes, DCs, and natural killer cells (Blank, Gajewski & Mackensen, 2005; Jin, Ahmed & Okazaki, 2011). When PD-L1 is litigated with its receptor, it has been seen to decrease T cell receptor-mediated proliferation and cytokine production, playing a role in tumor immune evasion (Blank, Gajewski & Mackensen, 2005). Immunotherapy that focuses on creating a PD-1/PD-L1 blockade has shown promising results as phase 1 and phase 2 trials in metastatic TNBC with a 5–42% response rate (Wein et al., 2018). Combining PD-L1 and ICOSL immunotherapy could prove to be of great benefit in the treatment of a variety of cancers, however, effects of such a novel treatment are currently unknown and further experiments are yet to be conducted.

As far as we know, there has not been much research investigating the molecular impact of ICOSL expression in subcellular sites other than the cell membrane, nucleus, and cytoplasm. Considering that ICOS is a transmembrane molecule, most of the recent study has been focusing on the roles of ICOSL expression on the plasma membrane (Aspord et al., 2013; Hutloff et al., 1999; Martin-Orozco et al., 2010). Our study is the first to show that ICOSL is not only expressed on the membrane, but also in the cytoplasm and nucleus of breast tumor cells. Whether ICOSL is also expressed in the other subcellular locations such as in the mitochondria remains but yet to be determined. It would be one of our future emerging tasks to understand the potential significance of ICOSL expressed in different subcellular sites.

## Conclusions

The results of the present study suggest that ICOSL is highly expressed in the tumors of patients with invasive breast carcinoma. The expression of ICOSL on cell membranes was associated with a worse prognosis in Luminal A/B, TNBC, and non-TNBC breast cancers. Thus, ICOSL expressed in cancer cells may be used as an independent prognosis factor for OS. Currently, treatment recommendations for sporadic breast cancers have not taken ICOSL expression into account as an important prognostic indicator. Our data provide an insight into the function of the ICOSL protein, facilitating development of novel therapies for sporadic breast cancers in the future.

## Supplemental Information

10.7717/peerj.6903/supp-1 Supplemental Information 1 Raw data collected from 562 patients with invasive breast cancer applied for data analyses and preparation for Fig. 3 and Tables 1–3.

## Abbreviations

AJCC: American Joint Committee on Cancer
APCs: antigen presenting cells
ER: estrogen receptor
HER2: human epidermal growth factor receptor 2
ICOS: inducible co-stimulator
ICOSL: inducible co-stimulator ligand
IHC: immunohistochemistry
OS: overall survival
PR: progesterone
Treg: regulatory T cells.

## Additional Information and Declarations

### Data Availability

The following information was supplied regarding data availability: The raw data are available in Dataset S1.
